# Are health risk attitude and general risk attitude associated with healthcare utilization, costs and working ability? Results from the German KORA FF4 cohort study

**DOI:** 10.1186/s13561-019-0243-9

**Published:** 2019-08-30

**Authors:** Johanna I. Lutter, Boglárka Szentes, Margarethe E. Wacker, Joachim Winter, Sebastian Wichert, Annette Peters, Rolf Holle, Reiner Leidl

**Affiliations:** 1 Helmholtz Zentrum München, German Research Center for Environmental Health (GmbH), Institute of Health Economics and Health Care Management, Ingolstaedter Landstr 1, 85764 Neuherberg, Germany; 2 Ludwig-Maximilians-Universität München, Institute for Medical Informatics, Biometry and Epidemiology, Marchioninistr 15, 81377 Munich, Germany; 3 Department of Economics, Ludwig-Maximilians-Universität München, Ludwigstr 33, 80539 Munich, Germany; 4 ifo Institute – Leibniz Institute for Economic Research at the University of Munich, Poschingerstr 5, 81679 Munich, Germany; 5 Helmholtz Zentrum München, German Research Center for Environmental Health (GmbH), Institute of Epidemiology, Ingolstaedter Landstr 1, 85764 Neuherberg, Germany; 6 Ludwig-Maximilians-Universität München, Munich Center of Health Sciences, Ludwigstr 28 RG, 80539 Munich, Germany

**Keywords:** Willingness to take risk, Healthcare utilization, Healthcare costs, Prevention, Population-based, Work absence

## Abstract

**Background:**

Risk attitudes influence decisions made under uncertainty. This paper investigates the association of risk attitudes with the utilization of preventive and general healthcare services, work absence and resulting costs to explore their contribution to the heterogeneity in utilization.

**Methods:**

Data of 1823 individuals (56.5 ± 9.5 years), participating in the German KORA FF4 population-based cohort study (2013/2014) were analyzed. Individuals’ general and health risk attitude were measured as willingness to take risk (WTTR) on 11-point scales. Utilization of preventive and medical services and work absence was assessed and annual costs were calculated from a societal perspective. Generalized linear models with log-link function (logistic, negative-binomial and gamma regression) adjusted for age, sex, and height were used to analyze the association of WTTR with the utilizations and costs.

**Results:**

Higher WTTR was significantly associated with lower healthcare utilization (physician visits, physical therapy, and medication intake), work absence days and indirect costs. Regarding preventive services, an overall negative correlation between WTTR and utilization was examined but this observation remained non-significant except for the outcome medical check-up. Here, higher WTTR was significantly associated with a lower probability of participation. For all associations mentioned, Odds Ratios ranged between 0.90 and 0.79, with *p* < 0.05. Comparing the two risk attitudes (general and regarding health) we obtained similar results regarding the directions of associations.

**Conclusions:**

We conclude that variations in risk attitudes contribute to the heterogeneity of healthcare utilization. Thus, knowledge of their associations with utilization might help to better understand individual decision-making – especially in case of participation in preventive services.

**Electronic supplementary material:**

The online version of this article (10.1186/s13561-019-0243-9) contains supplementary material, which is available to authorized users.

## Background

Risk attitude (RA) is a key determinant of decision-making. Especially medical decisions often involve a certain amount of risk and uncertainty, which is why research on RAs is of increasing importance within health economics.

Already intensively investigated in the economic setting, theoretical concepts, measurement techniques and determinants of RA have been developed and reported. Instruments to measure RA include lotteries, assessing hypothetical or actual behavior and self-reports based on situational questions and rating scales [1, 2].

According to previous studies, people’s attitude towards risk strongly depends on the specific setting, in which the decision has to be made. Thus, a person can have different RAs depending on whether he or she is faced with a financial decision, a decision or action concerning his or her health or any other domain [3]. Furthermore, several determinants of RAs have been identified. According to numerous corresponding reports, age and gender affect RAs with older people and women being more risk averse [4–7]. Dohmen et al. [8] reported height (the taller the more willing to take risks) and parental education (higher risk tolerance for higher parental education) as additional exogenous determinants. Thereafter, these two determinants were included in other studies to further investigate height and parental education as being exogenous determinants [9, 10].

Efforts have been undertaken to adapt the measuring instruments and concepts of RAs acquired in economic research and especially in decision theory to the health sector [11, 12]. In the context of health service research, previous studies analyzed the influence of RAs on health insurance demand [13], treatment choices [14, 15], behavioral health risks such as smoking, alcohol consumption and seat belt none-use [16] and physicians’ medical decision-making [17–19]. Decker et al. [20] analyzed the influence of health shocks on willingness to take risks and found a significant increase of risk aversion for those people who suffered from a health shock. This finding is in contrast with the previous assumption that RAs remain constant over lifetime [21].

Adding to the increasing importance of research on RAs in the field of health economics, this study addresses another open question concerning the role of RAs in context of demand for healthcare services. While there are some speculations in the literature, that RAs may affect the use of preventive services and medical care [16], the association is up to now unexamined. We aim to bridge this gap by analyzing the association of RAs with healthcare utilization and related costs. RA has been measured as self-reported willingness to take risk (WTTR) on 11-point scales as has been previously done by Dohmen et al. [8] [22], and further on by Decker et al. [20], Massin et al. [19], Van Der Pol et al. [23], and in a global study of economic preferences, by Falk et al. [24].

The paper has been organized according to the predefined hypotheses:

(i) Higher WTTR is associated with less participation in preventive services such as screening interventions and medical check-up programs, thus taking eventual health risks linked to late discovery of disease amenable to early intervention.
(ii) Higher WTTR is associated with lower general healthcare utilization and associated direct costs, thus taking eventual health risks linked to gaps in treatment initiation or adherence.

Following an explorative approach, we additionally examine the association of WTTR and indirect costs (work absence and early retirement) in a working-age sub cohort to fully investigate all components of disease related costs.

The directions of the hypotheses were further motivated by the general observation that healthcare utilization and costs increase with higher age, and by studies showing that higher age and risk tolerance are negatively correlated [5, 7, 25]. While there are some hints that RAs may vary in individuals over life time [20], we only take a cross-sectional view in this paper.

## Methods

### Data and study design

Data were taken from the population-based study KORA FF4 study (June 2013 to September 2014), the second follow-up of the KORA S4 study conducted in the city of Augsburg and two surrounding counties in southern Germany. Randomly drawn from the target population (adults aged 25–74 with German nationality) using population registries, 4261 subjects participated in the baseline survey S4 (1999–2001). Of those, 2279 participants aged 39 to 85 took part in the 14-year follow-up FF4 study. Detailed information about the study design, sampling methods, response rates and dropouts have been published elsewhere [26, 27]. Since only participants aged 73 and younger answered the RA questions, 428 (18.8%) participants were excluded from the present analysis. Furthermore, 28 observations had to be excluded due to missing data in the RA variables and variable height. Finally, data of 1823 participants aged 39 to 73 were included in the present cross-sectional analysis.

#### Ascertainment of risk attitudes

In order to measure RAs, participants were asked to rate their general willingness to take risk (G-WTTR) on an 11-point scale with 0 indicating ‘not at all willing to take risk’ and 10 ‘very willing to take risk’. Additionally, people’s willingness to take risk in six different domains of life (car driving, financial matters, sports and leisure, career, health, and faith in foreign people) was assessed using the same scale. The present analysis focuses on WTTR in general and regarding health (G-WTTR and H-WTTR) only, even though WTTR in relation to car driving or sports and leisure would also be conceivable in the context of health. The order of questions was chosen in accordance with the original version of the German Socio-Economic Panel (SOEP) where the G-WTTR is assessed first followed by the subdomains including H-WTTR. Dohmen et al. [8] confirmed the behavioral validity of this RA measurement technique by comparing the reported scale values to paid lottery choices acquired in a field experiment. To obtain comparable estimates for G-WTTR and H-WTTR in the regression analyses, we use a z-standardized version of the risk measures with mean = 0 and standard deviation (SD) = 1.

#### Measurement of covariates

Information on all covariates was collected in questionnaires or standardized interviews carried out by trained medical interviewers. Variables have been defined as follows: *Social class* by Helmert et al. [28] (an additive index of the variables ‘household net income’, ‘educational level’ and ‘occupational status’ that takes values between 1 and 27 with higher values indicating higher social class). *Comorbidity* (binary variable with 0: no comorbidity, 1: at least one of the following diseases: hypertension, diabetes, angina pectoris, stroke, cancer, which are known as prevalent diseases with high economic and patient relevant impact). *Smoking status* (current-, former- and never-smoker [29]). *Alcohol consumption* (binary variable with low risk: average daily alcohol intake ≤12 g for women and ≤ 24 g for men and elevated risk: average daily alcohol intake > 12 g for women and > 24 g for men [30]). *Physical activity* (binary variable with the characteristics active: regular sports in leisure time in summer and winter for ≥1 h per week, and inactive: < 1 h of sports per week).

### Assessment of preventive and medical service utilization

Participants were asked whether they have ever participated in a screening program (namely as programs to detect skin-, lung-, and colon cancer as well as breast- and cervical cancer for women and prostate cancer for men) or in a general medical check-up for the early identification of cardiovascular diseases.

Utilization of medical services was assessed using different time horizons with the last 7 days prior to the examination for use of pharmaceuticals, 3 months for the numbers of outpatient physician visits (subdivided into 15 groups of medical specialists excluding dentists [29]) and 12 months for hospital visits (numbers of outpatient hospital treatments and inpatient hospital days), visits to alternative practitioners, physical therapy treatments and rehabilitation stays. Assuming constant utilization, all data were extrapolated to 1 year in order to estimate general healthcare utilization within the last 12 months.

### Costs calculation

#### Direct costs

In Germany, costs of almost all healthcare services (except “out-of-pocket” expenditures e.g. for pharmacies) are covered by German statutory health insurance, which raises income-related insurance contributions. To estimate annual total direct medical costs, which represent a summary measure of the single healthcare utilization categories, we multiplied the reported utilizations with German unit costs (price year 2013) provided by Bock et al. [31]. An overview of all applied unit costs is available in Additional file 1: S1. Unit costs for physician visits varied between 19.36 € (for dermatologist) and 78.53 € (for psychotherapist) per contact. In case participants reported a physician visit in the previous 3 months without indicating the frequency (*n* = 2), one visit was imputed to follow a conservative approach.

Inpatient and outpatient hospital treatments were priced with 623.18 € and 46.80 € per day, respectively. We assessed 1408.22 € per day spent in the intensive care unit. For each day of inpatient rehabilitation we calculated 125.71 € and 62.36 € for outpatient rehabilitation. Costs for physical therapy treatment were rated with 17.04 € per visit. As Bock et al. [31] did not provide unit costs for alternative practitioner visits, costs were requested directly through the questionnaire. We imputed the average costs per visit (83 €), if participants stated an alternative practitioner visit without specifying resulting costs (*n* = 2).

The calculation of drug costs was restricted to prescription drugs only and based on information on name, pharmaceutical registration number and patient reported dosage of intake during the past week and combined with the pharmacy retail prices provided by the Scientific Institute of the AOK health insurance (WIdO [32]).

The calculation of direct annual costs did not include costs of preventive medical examinations.

#### Indirect costs

Annual indirect costs were calculated for all participants with employable age 65 and younger. Productivity losses due to early retirement and work absence days (only for those with regular full- or part-time employment) were taken into account to calculate indirect costs from a societal perspective using the human capital approach [33, 34]. According to this approach, a year of disability is valued with the average labor costs, provided by the Federal Statistical Office [35] (35.904 € in 2013).

Early retirement was considered for those who reported retirement due to health or other reasons. To assess costs caused by temporary work absence, participants were asked how many days they had been absent due to illness during the previous 12 months. Values greater than 208 days (number of actual working days in 2013 in Germany [36]) were corrected to 208 (*n* = 3). Each day of absence was valued with 172.45 €, the quotient of the average labor costs and the actual working days.

### Statistical analysis

Unadjusted means for utilizations and costs as well as the histograms of the two risk measures G-WTTR and H-WTTR were calculated. Additionally, the correlation coefficient of G-WTTR and H-WTTR was assessed using Pearson’s correlation coefficient.

All regression models were performed separately for the two RA measures using the same functions and covariates to identify potential differences and similarities in the effect estimates of RAs on preventive and medical services and costs. Since we used the z-standardized version of the RA values in the regression analyses, all estimates can be interpreted as the effect on the outcome for a one SD increase in the independent variables G-WTTR and H-WTTR .

In a first step, logistic regression models were applied to analyze the association of RAs with healthcare utilization and work absence. Second, participants who reported values greater than zero (users only) were then included in a generalized linear model with a zero-truncated negative binomial distribution and log-link function to evaluate the association between RAs and frequency of utilization. Finally, to examine the association of RAs with direct medical and indirect costs, we fitted gamma regression models with log-link functions using the procedure of generalized regression models (GLM). Use of this model was necessary to meet the demands of the typically skewed distribution of costs. We imputed a value of 1 € for all participants with zero direct (*n* = 289, 15.9%) and indirect (*n* = 735, 51.4%) costs as recommended by Barber et al. [37]. In line with previous literature, all models were adjusted for the exogenous determinants age, sex, and height [8].

Significance levels were set at the 5% level. Statistical analyses have been carried out with SAS software V.9.3 (SAS Institute, Cary, North Carolina, USA).

### Sensitivity analysis

We performed a sensitivity analysis to identify the influence of the covariates included in the regression models. Anderson et al. [16] reported significant positive associations between risk aversion and behavioral health risks (smoking, alcohol consumption, and being overweight). Therefore, we extended the basic model by adding the following variables, which we considered to be additional potential confounders of RA and the outcomes of interest: social class, comorbidity, smoking status, alcohol consumption and physical activity.

## Results

Table 1 presents the socio-demographic characteristics of the study sample. In the sample population, 52.6% of participants were female, the mean age was 56.5 (SD 9.5) years and mean height was 169.7 (SD 9.5) cm. Total mean annual direct costs were 1873 € (SD 6026) per participant. Mean annual indirect costs, which were only calculated for participants with employable age ≤ 65 were 3938 € (SD 10031) and were predominantly caused by early retirement (74%). Overall, 15.9% of all participants had no direct medical costs, whereas roughly half of the participants with employable age incurred no indirect costs in 2013. Detailed information on the mean frequency of utilization and the adapted unit costs for each direct and indirect cost category are provided in Additional file 1: S1.

**Table 1:** Socio-demographic characteristics of the KORA FF4 sub-sample

Characteristics	Total (*n* = 1823)
Male	864 (47.4)
Age (years)	56.5 ± 9.5
Height (cm)	169.7 ± 9.5
Social class index	15.4 ± 5.0
Physical Activity	
Active (≥ 1 h of sports per week)	1097 (60.2)
Inactive (< 1 h of sports per week)	726 (39.8)
Alcohol consumption	
Low risk (women ≤12 g, men ≤24 g p.d.)	1267 (69.5)
Elevated risk (women > 12 g, men > 24 g p.d.)	555 (30.5)
Smoking status	
Current smoker	328 (18.0)
Former smoker	708 (38.8)
Never smoker	787 (43.2)
Hypertension ^a^	580 (31.9)
Diabetes mellitus ^b^	121 (6.6)
Angina Pectoris	134 (7.4)
Stroke	28 (1.5)
Cancer	158 (8.7)
Risk attitude	
G-WTTR	4.46 ± 2.21
H-WTTR	3.14 ± 2.18
Annual costs (€)	
Direct costs	1873 ± 6026
Indirect costs ^c^	3938 ± 10,031

data are n (%) or mean ± SD

G-WTTR: Willingness to take risk in general; H-WTTR: Willingness to take risk regarding health

^a^*n* = 1821: two observations with missing value

^b^*n* = 1819: four observations with missing value

^c^*n* = 1429: indirect costs only for persons with employable age ≤ 65 years

### Descriptive analysis of the risk attitude variables

**Fig. 1:**
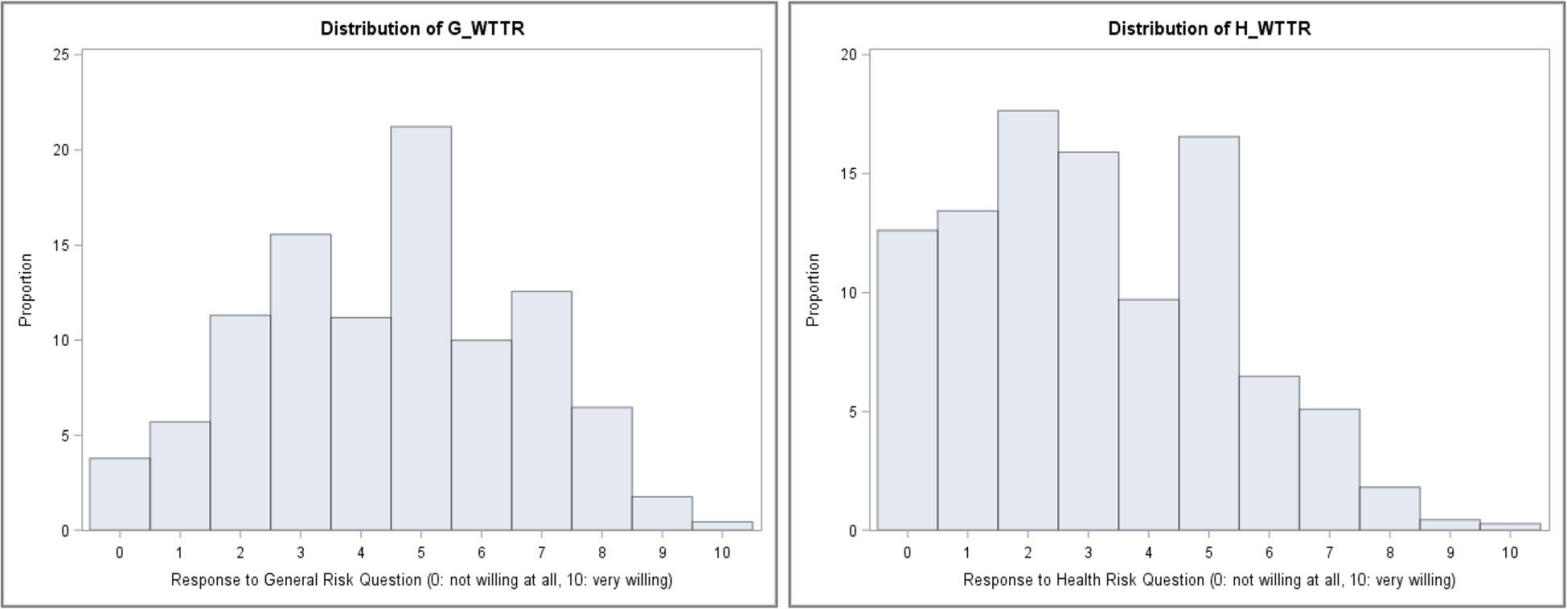
Histogram of responses of G-WTTR and H-WTTR measured on an 11-point scale

The distributions of the two RA measures are displayed in Fig. 1. Mean values were 4.46 (SD 2.21) for G-WTTR and 3.14 (SD 2.18) for H-WTTR. The highest possible number 10 was chosen by less than 1 % of all participants. This was true for both RA measures. Looking at the other side of the scale, roughly 4% stated a value of 0 for G-WTTR, and a much larger proportion of participants (12.6%) choose the smallest value on the scale to indicate their H-WTTR. The values 5 and 2 were the modal responses of general and health WTTR, respectively. Pearson’s correlation coefficient revealed a moderate positive linear relationship between the two measures with r = 0.40 (p. < 0001). When adjusting for age and sex, the partial correlation coefficient remained almost unchanged with r = 0.37 (p. < 0001).

### Preventive services

The odds ratios (OR) of general and health WTTR for the likelihood of utilizing preventive services are summarized in Table 2. Each effect estimate is based on a separate regression analysis with the dependent variable in the left column and either G-WTTR or H-WTTR as part of the regression function. We found that the probability of ever undergoing a medical check-up decreased with higher H-WTTR (OR 0.89, 95% confidence interval (CI) 0.81–0.98). Considering all types of screening programs, women had a 9.55 times (general) or 9.67 times (health) higher odds to have ever participated in a screening program compared to men. When limiting the screening programs to skin cancer and colon cancer, which are practicable for both sexes, the effect estimates decrease but remain significant with women having had a 1.71 (general) or 1.67 (health) times higher probability of screening participation compared to men. Higher age was associated with a higher probability of participation except in the case of cervical cancer screening. Whereas the estimates for sex and age were significant in nearly all analyses, only a few significant associations between height and the utilization of preventive services were observed with the trend of higher probabilities for taller participants.

**Table 2:** Probability of using preventive services (ever) Logistic regression models, adjusted for age, sex and height

Dependent variable	Proportion (yes)	G-WTTR	H-WTTR
		OR [95% CI]	OR [95% CI]
Medical check-up	56.6%	1.02 [0.93–1.12]	0.89* [0.81–0.98]
Screening (overall)	88.5%	0.91 [0.77–1.06]	0.94 [0.81–1.09]
Skin cancer	58.5%	1.03 [0.93–1.13]	0.95 [0.86–1.04]
Colon cancer	61.0%	0.95 [0.85–1.05]	0.93 [0.84–1.03]
Breast cancer ^a^	78.6%	0.92 [0.77–1.10]	0.95 [0.80–1.13]
Cervical cancer ^a^	84.7%	1.01 [0.84–1.21]	0.90 [0.75–1.08]
Prostate cancer ^b^	60.5%	0.96 [0.82–1.12]	0.87 [0.75–1.01]

**significant at the 1% level/ * significant at the 5% level

G-WTTR: Willingness to take risk in general; H-WTTR: Willingness to take risk regarding health

^a^Only for women (*n* = 959 observations), adjusted for age and height

^b^Only for men (*n* = 864 observations), adjusted for age and height

### Medical healthcare utilization

Table 3 displays the ORs for the association of RAs with the probability of having any healthcare utilization, work absence days or early retirement. We found a significant association between H-WTTR and the probability of having at least one physician visit in the previous 3 months with an OR of 0.90 [95% CI 0.81–0.99] for an increase in one SD in H-WTTR. A similar association for G-WTTR with trend *p* < 0.10 was observed. Higher G-WTTR was associated with a decreasing likelihood of medication intake (OR 0.82, 95% CI 0.74–0.91). The majority of ORs for the variable sex showed values greater than 1 indicating higher probabilities of utilization for women compared to men. We found positive associations between age and utilizations. We did not find significant associations between height and the outcomes except in the case of pharmaceutical use (OR 0.98, 95% CI 0.97–1.00 for G-WTTR and H-WTTR).

**Table 3:** Probability of using medical services Logistic regression models, adjusted for age, sex and height

Dependent variable		Proportion (yes)	G-WTTR	H-WTTR
			OR [95% CI]	OR [95% CI]
Physician visit	(3 months)	65.6%	0.91 [0.82–1.01]	0.90* [0.81–0.99]
Hospital treatment	(12 months)	19.8%	1.01 [0.88–1.16]	1.00 [0.87–1.14]
Rehabilitation	(12 months)	4.6%	0.85 [0.67–1.07]	0.99 [0.79–1.23]
Physical therapy	(12 months)	32.6%	0.96 [0.87–1.06]	0.89* [0.81–0.99]
Alternative Physician	(12 months)	9.9%	0.96 [0.82–1.13]	0.93 [0.79–1.09]
Medication intake	(7 days)	62.3%	0.82** [0.74–0.91]	0.95 [0.86–1.05]

**significant at the 1% level/ * significant at the 5% level

G-WTTR: Willingness to take risk in general; H-WTTR: Willingness to take risk regarding health

The results of the zero-truncated negative-binomial regressions describing the associations between RAs and the frequencies of utilization given any utilization are summarized in Table 4. We found that an increase of one SD in G-WTTR lead to a 6% decrease in the number of physician visits among participants with at least one visit during the period examined. Among participants who reported an inpatient hospital stay, higher H-WTTR led to an increase in the number of inpatient hospital days (OR 1.15, 95% CI 1.02–1.30). A one SD increase in G-WTTR was associated with a 1.32 (95% CI 1.16–1.51) times higher number of alternative physician visits.

**Table 4:** Frequencies of utilization (users-only) Zero truncated negative-binomial regression models, adjusted for age, sex and height

Dependent variable	Users	Frequency of utilization if used	G-WTTR	H-WTTR
Number of	(n)	(mean, unadjusted)	exp(estimate) [95% CI]	exp(estimate) [95% CI]
Physician visits	1195	3.9 (3.9)	0.94* [0.90–0.99]	0.99 [0.94–1.03]
Inpatient hospital days	253	9.3 (19.9)	1.05 [0.92–1.20]	1.15* [1.02–1.30]
Rehabilitation days	84	24.5 (19.3)	0.92 [0.81–1.04]	0.95 [0.84–1.07]
Physical therapies	594	16.5 (21.1)	0.96 [0.89–1.03]	1.05 [0.98–1.13]
Alternative physician visits	180	4.2 (5.4)	1.32** [1.16–1.51]	1.10 [0.97–1.25]
Pharmaceuticals	1135	2.6 (2.0)	0.97 [0.93–1.02]	0.97 [0.93–1.01]

**significant at the 1% level/ * significant at the 5% level

G-WTTR: Willingness to take risk in general; H-WTTR: Willingness to take risk regarding health

### Work absence days and early retirement

Work absence days were reported by 54% of the 1079 individuals with full-time or part-time employment and 8% retired early with age ≤ 65 years (see Table 5). Individuals with higher general or health WTTR were less likely to have at least one work absence day (OR 0.88, 95% CI 0.77–0.99 for G-WTTR and OR 0.88, 95% CI 0.78–0.99 for H-WTTR). The number of work absence days significantly decreased by 20% for a SD deviation increase in G-WTTR. We investigated a similar association between the number of work absence days and H-WTTR (OR 0.85, 95% CI 0.77–0.94).

**Table 5:** Work absence and early retirement (sub-sample including working-age participants only)

Dependent variable		G-WTTR	H-WTTR
	Proportion yes / Mean frequency	exp(estimate) [95% CI]	exp(estimate) [95% CI]
Probability of using medical services (logistic regression)			
Work absence ^a^	53.6%	0.88* [0.77–0.99]	0.88* [0.78–1.00]
Early retirement ^b^	8.1%	0.78 [0.44–1.63]	0.74 [0.42–1.30]
Frequencies of utilization ^c^ (negative-binomial regression)			
Work absence days	14.7 (27.1)	0.80** [0.73–0.88]	0.85** [0.77–0.94]

**significant at the 1% level/ * significant at the 5% level

G-WTTR: Willingness to take risk in general; H-WTTR: Willingness to take risk regarding health

^a^*n* = 1079: work absence only for full-time and regular part-time employees

^b^*n* = 138: early retirement only for pensioners with age ≤ 65 years

^c^*n* = 578

### Annual direct and indirect costs

The associations of general and health WTTR with total annual direct and indirect costs are summarized in Table 6. We observed a negative association of G-WTTR on total indirect costs expressed by an estimate of 0.79 (95% CI 0.69–0.90) for a one SD increase in G-WTTR. We did not find significant associations between RAs and the outcome direct costs. However, a trend was visible, indicating higher direct costs with increasing H-WTTR. Regarding the covariates included in the gamma regression, an older age was associated with an increase in costs, whereas bigger height was associated with decreased costs.

**Table 6:** Annual direct medical and indirect costs Gamma regression models, adjusted for age, sex and height

	G-WTTR	H-WTTR
	exp(estimate) [95% CI]	exp(estimate) [95% CI]
Total direct medical costs	1.00 [0.92–1.09]	1.06 [0.98–1.15]
Total indirect costs ^a^	0.79** [0.69–0.90]	0.91 [0.80–1.03]

**significant at the 1% level/ * significant at the 5% level

G-WTTR: Willingness to take risk in general; H-WTTR: Willingness to take risk regarding health

^a^*n* = 1429: indirect costs only for persons with employable age ≤ 65 years

1€ was assigned to observations with costs = 0

### Results of the sensitivity analysis

Estimates of the associations between the RAs and the likelihood of participating in a screening intervention or medical check-up remained unchanged when adapting the extended model, which included additional lifestyle and disease-specific variables as potential confounders. Gamma regressions for direct and indirect costs were performed using the same model. Similar to the results obtained from the small model, higher G-WTTR was associated with lower indirect costs. In addition, we observed a trend with *p* < 0.10 concerning H-WTTR and total direct medical costs: a one SD increase of H-WTTR was associated with 1.07 (0.99–1.16) times higher total direct costs.

## Discussion

This cross-sectional analysis of data from the population-based KORA FF4 sample evaluated the association of RAs with the utilization of preventive and medical services as well as direct medical and indirect costs. RA was analyzed for both, willingness to take risk in general, and willingness to take risk regarding health. First, our results indicated a negative but mainly non-significant correlation between higher WTTR and participation in screening programs and preventive check-ups. Second, we found that individuals with higher WTTR were less likely to use the following healthcare services: physician visits, physical therapy (only for H-WTTR), and medication intake (only for G-WTTR). Finally, higher WTTR was associated with fewer work absence days.

The same set of RA questions has been included in previous waves of the German Socio-Economic Panel (SOEP), a representative panel survey of the resident adult population of Germany with approximately 11,000 private households and 22.019 individuals [38]. Based on this data, Dohmen et al. [8] reported mean values of 4.42 (SD 2.38) and 2.93 (SD 2.47) for general and health WTTR, respectively. As the paper focusses on the general measure, detailed information about the distribution of answers is only available for this risk measure. Similar to our results, the most frequent answer was 5 and roughly 7% of the SOEP participants chose the smallest possible number 0. This similarity of results underlines the representativeness of the descriptive findings of the present analysis.

### Preventive services

Turning to our results for the utilization of preventive services, our data could not confirm, in terms of statistically significant estimates, our hypothesis that higher WTTR is negatively correlated with participation in general. Only in the case of medical check-up, we found an increase in H-WTTR associated with an 11% decrease in the likelihood of ever having participated in such a check-up.

Comparison with prior research is limited as, to the best of our knowledge, this is the first study to analyze RAs in association with preventive services. The non-significant trend to a negative association found in general suggests that further tests of the hypothesis in other study settings with detailed data on preventive services seem warranted. In the present study, participation rates were found to be very high with nearly 90% reported participation in at least one screening intervention and above 60% for the single screening programs. Thus, voluntary participation in our study may have increased the selection of individuals specifically interested in health issues and preventive services.

### Healthcare utilization

Our study hypothesis was that higher WTTR is associated with less healthcare utilization, featuring individuals less concerned with their health and possibly necessary care. The finding that higher WTTR was associated with a lower probability of having had a physician visit and a smaller number of physician visits given any reported utilization supports this. However, one might also consider a mechanism pointing in the opposite direction: Because of risky behavior, risk tolerant individuals might need medical treatment more often. Investigating this notion, we found higher H-WTTR to be associated with a higher number of hospital days given at least one hospital stay. While hospital diagnoses might help to identify relevant cases such admissions as a result of accidents, lack of respective data in our study limited further confirmation of this notion. Since a survey of historical risk attitude in a case-control design hardly seems possible, large cohort studies would be needed to test this notion.

Regarding alternative medicine, we found a positive association between higher G-WTTR and the number of alternative physician visits. This finding aligns with Sturm et al. [39], who evaluated the association of self-assessed risk seeking attitudes and the utilization of alternative medicine. Participants who considered themselves as more risk taking than the average person had a 2.47 times higher chance of visiting an alternative medicine provider.

Based on previous literature reporting correlations between WTTR and exogenous determinants, we included age, gender, and height as potential confounding variables in our analyses. As expected, we could investigate higher probabilities of utilization for older age. Interestingly, there was no significant association between age and the frequency of utilization given at least one reported utilization with the exception of use of pharmaceuticals. Here, older age was significantly associated with a greater number of pharmaceuticals used.

Although we could not observe significant associations between height and the probability or frequency of utilization, we found that bigger height was associated with lower total direct medical costs. A possible explanation might be an association between lower body height and elevated risk for cardiovascular disease and types of cancer, thus leading to increased healthcare costs [40, 41].

### Work absence

To observe the entirety of sickness costs and consequential costs, we complemented the analyses of direct healthcare cost (e.g. physician visits and hospital stays) by components of indirect costs, namely work absence and early retirement. There is evidence that risk seeking individuals are more likely to become an entrepreneur, meaning that people with high RA are more likely to work on a self-employed basis [42, 43]. We assume this to be a possible explanation for our finding that higher RA was associated with a lower probability of having work absence days and also lower indirect costs. A second explanation could be that individuals with higher RA still attend to work even if they are not absolutely healthy. The extra risk regarding a possible aggravation of the state of health is accepted in this case. Thirdly, the specific type of occupation and the associated levels of physical activity have to be considered when interpreting these results. It is, of course, more difficult to show up for a physically stressful job when feeling sick than for a desk job. Application of the extended model to the cost regression analysis did not affect the estimates of RAs. This underlines the robustness of our results even when adjusting for additional variables such as socio economic status, lifestyle factors and the presence of certain diseases.

### General risk attitude or health risk attitude

Dohmen et al. [8] identified the general risk question as the best “all round” predictor to measure people’s willingness to take risks. However, the authors state that the domain-specific RAs should be preferred in the corresponding domains. For example, health RA is the best predictor for assessing risky health behavior such as smoking. This is also supported by Massin et al. [19], who studied the association of general practitioner’s (GP) RA and their medical practices. The authors present a comparison of scales and lotteries as different measures for RA regarding the predictive power of the tools on GP’s medical practices. A slightly modified version (addition of the word “daily”) of the presented 11-point scale was also included in the comparison. The authors conclude that the general measure is not suitable in predicting GP’s medical practices and the domain-specific measures are to be preferred. Accordingly, we expected H-WTTR to be the best measure to analyze the association of RA with healthcare utilization and related costs. This was not confirmed by our results as the estimates for general and health WTTR only slightly differed in the effect size.

### Strengths and limitations

To the best of our knowledge, this is the first study to analyze preventive and medical healthcare utilization, work absence and costs in association with individuals’ RAs. We make use of a simple risk measure, which can be easily captured through questionnaires. In this way assessed RAs values represent actual behavior in paid lottery choice experiments very well, as shown by Dohmen et al. [8]. The study provides a comprehensive overview of the direct and indirect cost components and sheds light on the specific healthcare services whose degree of utilization is associated with individuals’ RAs.

Several limitations need to be noted regarding the present study. Individuals’ RAs were assessed at a single point in time. Therefore, it cannot be ruled out, that important events in the past may have influenced the RAs in either direction. Decker et al. [20] provide an overview of articles studying important events, which were found to influence RA. Additionally, Liebenehm et al. [44] and Sachs et al. [45] report changes in RAs over time and therefore advocate the time-variability of RAs. However, for the purpose of our study, which was to identify aspects of healthcare utilization associated with RAs in cross-section, the results should not be biased by the assumption. It should further be noted, that inclusion of the variable “parental education” as a potential confounder in the regression analyses was not possible as it was not assessed in the KORA FF4 study or any previous survey. Nevertheless, our results remain the same when adjusting for the variable social status, which might have a high correlation with parental education. Regarding the utilization of preventive services, attitudes to health risks caused due to the screening intervention (e.g. post-colonoscopy complications [46]) were not considered or included in the analyses and might be an additional factor influencing the decision whether to undergo a screening procedure.

The cross-sectional study design implies further limitations. Healthcare utilization was assessed by asking participants to provide information retrospectively. This method is generally seen to be vulnerable to recall bias. By adapting the abridged time horizons for the specific healthcare categories (1 week for pharmaceuticals, 3 months for physician visits and 12 months for hospital stays and rehabilitations), we tried to circumvent this problem, as recommended by Seidl et al. [47]. Furthermore, results may be biased by the composition of the study sample, as the FF4 study is the second follow-up of the baseline S4 study. Participation rates vary with 66% for S4, 80% for the second follow-up F4 and 69% for the present FF4 study. This so-called selection bias cannot be ruled out and is a general limitation of studies with voluntary participation. When interpreting results, the restricted generalizability has to be kept in mind.

## Conclusions

In conclusion, we observed associations between RAs and the likelihood and frequencies of utilizing specific healthcare services in the KORA FF4 subsample and an overall trend indicating a negative correlation between higher WTTR and the participation in preventive services. Further research should be undertaken to analyze the assumption of the time stability of RAs. Therefore, longitudinal surveys with multiple assessments of RAs are necessary to evaluate possible long-term changes. From a methodological point of view, our results indicate that both RA measures, G-WTTR and H-WTTR, seem to be useful when analyzing RAs in association with healthcare utilization, and results do not differ considerably depending on the chosen measure. From a societal perspective, our results indicate that RAs explain part of the heterogeneity of healthcare utilization. Regarding preventive programs, our findings indicate that interventions such as information campaigns intended to increase participation rates in screening programs and medical check-ups might be more effective when targeted at specific RA groups. Overall, our results contribute to the aim of better understanding individual health decisions.

## Additional file

Additional file 1: **S1.** Utilization of healthcare services and unit costs. (DOC 60 kb)

## Data Availability

The full dataset supporting the conclusions of this article is available upon request and application from the Cooperative Health Research in the Region Augsburg (KORA; https://www.helmholtz-muenchen.de/kora/ueber-kora/index.html).

## Abbreviations

CI: Confidence Interval
H-WTTR: Willingness to take risk regarding health
GLM: Generalized Linear Model
GP: General Practitioner
G-WWTR: Willingness to take risk in general
KORA: Cooperative Health Research in the Augsburg Region
OR: Odds Ratio
RA: Risk attitude
SD: Standard Deviation
SOEP: The German Socio-Economic Panel
WIDO: AOK Research Institute
WTTR: Willingness to take risk
