# Supplementary material for: Are health risk attitude and general risk attitude associated with healthcare utilization, costs and working ability? Results from the German KORA FF4 cohort study
Source: Health Econ Rev. 2019 Aug 30;9:26. doi: 10.1186/s13561-019-0243-9 (PMC6734302; doi:10.1186/s13561-019-0243-9)
Supplement: Supplementary file 1 — S1. Utilization of healthcare services and unit costs. (DOC 60 kb) [file 13561_2019_243_MOESM1_ESM.doc]

**Additional file**

| **Supplement S1** Utilization of healthcare services and unit costs | | | | | | | | |
| --- | --- | --- | --- | --- | --- | --- | --- | --- |
|  | | Participants using resource | | | Frequency of  utilization  (if used) | | | Unit costs  per day / visit  (Bock et al. 2015) ^a^ |
|  |  | n | % | | mean | SD | in € (2013) | |
| **Direct cost categories** | | | | | | | | |
| Physician visits in total (3 months) | | 1195 | 65.6 | | 3.9 | 3.9 |  | |
|  | General practitioner | 855 | 46.9 | | 1.9 | 1.7 | 20.57 | |
|  | GP for internal medicine | 210 | 46.9 | | 1.8 | 1.4 | 20.57 | |
|  | Specialist for internal medicine | 146 | 8.0 | | 1.8 | 2.8 | 63.53 | |
|  | Gynaecologist | 184 | 10.1 | | 1.1 | 0.4 | 31.27 | |
|  | Ophthalmologist | 196 | 10.8 | | 1.3 | 1.0 | 36.55 | |
|  | Orthopaedist | 269 | 14.8 | | 2.0 | 1.5 | 25.53 | |
|  | Otorhinolaryngologist | 104 | 5.7 | | 1.6 | 1.2 | 27.80 | |
|  | Surgeon | 51 | 2.8 | | 2.1 | 1.9 | 44.09 | |
|  | Dermatologist | 159 | 8.7 | | 1.6 | 1.0 | 19.36 | |
|  | Radiologist | 138 | 7.6 | | 1.5 | 3.2 | 46.80 | |
|  | Urologist | 89 | 4.9 | | 1.2 | 0.4 | 24.91 | |
|  | Neurologist / psychiatrist | 91 | 5.0 | | 1.4 | 0.9 | 46.49 | |
|  | Psychotherapist | 51 | 2.8 | | 4.7 | 4.4 | 78.53 | |
|  | Specialist in occupational medicine | 35 | 1.9 | | 1.1 | 0.2 | 20.57 | |
|  | Other physician | 54 | 3.0 | | 2.2 | 5.3 | 46.80 | |
| Hospital treatment (12 months) | | | | | | | | |
|  | Inpatient hospital treatment | 253 | 13.9 | | 9.3 | 19.9 | 623.18 | |
|  | Intensive care unit | 38 | 2.1 | | 2.5 | 2.3 | 1408.22 | |
|  | Outpatient hospital treatment | 108 | 5.9 | | 2.0 | 5.2 | 46.80 | |
| Rehabilitation (12 months) | | | | | | | | |
|  | Inpatient rehabilitation | 50 | 2.7 | | 27.0 | 18.7 | 125.71 | |
|  | Outpatient rehabilitation | 34 | 1.9 | | 20.9 | 20.0 | 62.36 | |
| Physical therapy (12 months) ^b^ | | 594 | 32.6 | | 16.5 | 21.1 | 17.04 | |
| Alternative physicians (12 months) | | 180 | 9.9 | | 4.2 | 5.4 | Self-reported costs | |
| Pharmaceuticals | | 1135 | 62.3 | | 2.6 | 2.0 | AOK Institute | |
| **Indirect cost categories ^c^** | | | | | | | | |
| Work absence ^d^ (number of days, 12 months) | | 578 | | 31.7 | 14.7 | 27.1 | 172.45 per day | |
| Early retirement ^e^ | | 116 | | 6.4 | - | - | 35.904 per year | |

^a^ provided by Bock, J., Brettschneider, C., Seidl, H., Bowles, D., Holle, R., Greiner, W., König, H.: Calculation of standardised unit costs from a societal perspective for health economic evaluation. Gesundheitswesen (Bundesverband der Arzte des Offentlichen Gesundheitsdienstes (Germany)) 77(1), 53-61 (2015).

^b^ n=1822: one observations with missing information on physical therapy

^c^ n=1429: indirect costs only for persons with employable age ≤ 65 years

^d^ n=1079: work absence only for full-time and regularly part-time employees

^e^ n=138: early retirement only for pensioners with age ≤ 65 years
